# The multifaceted role of pirfenidone and its novel targets

**DOI:** 10.1186/1755-1536-3-16

**Published:** 2010-09-01

**Authors:** José Macías-Barragán, Ana Sandoval-Rodríguez, Jose Navarro-Partida, Juan Armendáriz-Borunda

**Affiliations:** 1 Institute for Molecular Biology and Gene Therapy, Department of Molecular Biology and Genomics, University of Guadalajara, Guadalajara, Mexico; 2 O.P.D. Hospital Civil de Guadalajara, Guadalajara, Mexico

## Abstract

**Background:**

Pirfenidone (PFD) is a molecule that exhibits antifibrotic properties in a variety of *in vitro *and animal models of lung, liver and renal fibrosis. These pathologies share many fibrogenic pathways with an abnormal fibrous wound-healing process; consequently, tissue repair and tissue regeneration-regulating mechanisms are altered.

**Objective:**

To investigate the usefulness of PFD as an antifibrotic agent in clinical and experimental models of fibrotic disease.

**Conclusions:**

There is a growing understanding of the molecular effects of PFD on the wound healing mechanism, leading to novel approaches for the management of fibrosis in lung, liver and renal tissues. Although the optimum treatment for fibrosis remains undefined, it is possible that combined therapeutic regimens that include this wide-application molecule, pirfenidone, could offer a useful treatment for fibrotic disease.

## Introduction

**Figure 1:**
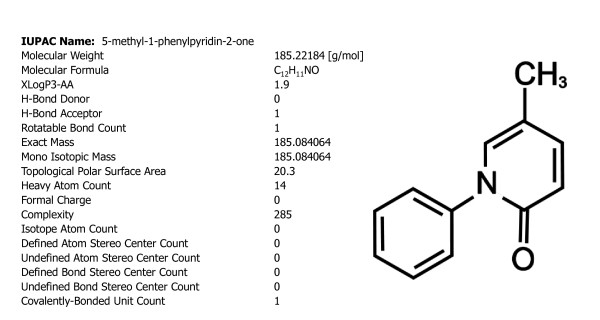
**Chemical characteristics of the pirfenidone molecule**.

Pirfenidone (PFD) is a pyridine(5-methyl-1-phenyl-2-(1H)-pyridone) with a simple chemical structure (Figure 1), which was initially developed as an antihelminthic and antipyretic agent [1]. PFD is very soluble in alcohol and chloroform; in aqueous solutions, the maximum concentration is 2%. The PFD molecule is able to move through cell membranes without requiring a receptor. When administered orally, PFD is easily absorbed in the gastrointestinal (GI) tract, reaching most tissues and crossing the blood-brain barrier. After oral administration, PFD reaches its maximum levels in blood after 1 to 2 hours and is almost fully eliminated in urine after another 6 hours. Regarding its safety, most studies have reported no significant toxicity attributable to the drug at doses of around 2500 mg/day; minor side effects observed include nausea, photosensitivity and GI issues.

## Pharmacokinetics of PFD in human and animal models

Studies on the pharmacokinetics and metabolism of PFD conducted by Shi *et al *[2] evaluated the pharmacokinetics of single and multiple oral doses of PFD in healthy adults in a randomized, dose-escalating study. The drug was rapidly absorbed (t_max _= 0.33 to 1 hours) and cleared (t_1/2 _= 2 to 2.5 hours). Pharmacokinetic parameters after multiple doses were similar to those after single doses, and concomitant intake of food reduced by 20% the rate and extent of absorption, which are associated with better tolerability of PFD. No significant sex differences were noted for the pharmacokinetic variables. Rubino *et al *[3] reported decreased rate of PFD absorption in adults when the drug was given with food. Analysis of adverse events (AEs) revealed a correlation between PFD C_max _and the risk of AEs associated with the GI system, suggesting that food may reduce the risk of certain AEs associated with PFD administration, which may improve tolerability. In mice, Cho *et al *[4] observed that plasma PFD levels fell rapidly, with a mean residence time of 6.3 min, which agrees with the rapid disappearance of the drug. The volume of distribution at steady state (Vd_ss_) was 0.71 ml/g, indicating that moderate extravascular distribution occurred within 5 min, with the drug reaching the following areas in descending order: kidney, liver, ventricle, lung, spleen, pancreas, testes, GI system, brain, skeletal muscle, adrenal glands and epididymal fat pad. Two metabolites were identified, which seem to be produced from oxidation of the methyl group on the pyrilidone ring followed by subsequent formation of the carboxylic acid.

Braim [5] observed mild and temporal effects during PFD administration in horses, including tachycardia and muscle fasciculations, effects that stopped when the infusion ended. By the end of the 5 min infusion, PFD and its two main metabolites, hydroxypirfenidone and carboxypirfenidone, were detected, and mean peak plasma concentration of PFD was 182.5 μmol/L. Forty minutes after infusion, mean peak plasma concentrations of hydroxypirfenidone and carboxypirfenidone were 1.07 and 3.4 μmol/L respectively. No parent drug or metabolites were detected at 24 hours.

In a sheep model, Bruss *et al *[6], observed that plasma PFD disappeared in accordance with first order kinetics with a clearance of 1.2 l/kg/h, a half-life of 24 min and distribution volume of 0.71 l/kg. After 48 h, the largest quantities were found in lungs, liver and the intestinal wall. In addition, the main metabolites were found in plasma and urine (half-life of 44 min); additional metabolites found in urine were hydroxypirfenidone glucuronide and acetoxypirfenidone. Approximately 80% of the tracer eventually appeared in the urine, of which approximately 50% was in the form of identifiable metabolites. Less than 1% of the dose appeared in the urine in the form of the parent drug. Quantitatively, most of the metabolites appeared in the urine within 2 h. Thus, these pharmacokinetic results support a tied regimen of PFD in patients with fibrotic disease.

PFD has been tested in a variety of cellular and animal models of inflammation and fibrosis, and has been shown to hav eanti-inflammatory, antioxidative stress and antiproliferative properties. PFD is known to regulate key fibrotic cytokines and growth factors. It inhibits several inflammatory mediators, has an antioxidant effect, and restores immune response balance. Beneficial effects have been shown for PFD in the treatment of fibrotic disease, including renal, liver and pulmonary fibrosis and multiple sclerosis (MS), conditions that share the pathology of abnormal deposition of collagen, which is determinant of clinical outcome. In these fibrosis-related diseases, the amount of collagen deposited in the tissue is controlled by the balance between synthesis (regulated at the transcriptional and translational level) and degradation of collagen in extracellular matrix (ECM) by matrix metalloproteinases (MMPs), which are regulated by tissue inhibitors of metalloproteinases (TIMPs). In fibrosis, the positive balance to collagen synthesis is influenced by production of transforming growth factor TGFβ and other growth factors, which can be downregulated by PFD.

Currently, several clinical trials using PFD for various diseases have been completed; these include studies on pulmonary fibrosis associated with Hermansk-Pudlak syndrome, focal segmental glomerulosclerosis, idiopathic pulmonary fibrosis, hypertrophic cardiomyopathy, kidney disease in patients with diabetes, neurofibromatosis type 1, plexiform neurofibromas, and fibrosis caused by radiation therapy for cancer. In addition, an open-label study on the long-term safety of PFD in patients with idiopathic pulmonary fibrosis has been devised, and there are studies investigating use of PFD as a non-invasive treatment for uterine leiomyomas, its influence on heart function and exercise capacity in patients with hypertrophic cardiomyopathy, its usefulness as a permeability factor in focal segmental glomerulosclerosis [1], and its benefits as a treatment for hypertrophic scars caused by burns in pediatric patients (Armendariz-Borunda *et al*., unpublished data).

**Figure 2:**
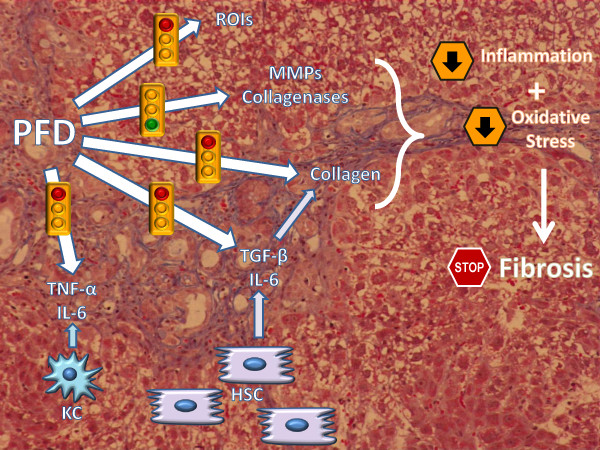
**Molecular mechanisms of pirfenidone in the reduction in fibrosis**. Green indicates the mechanisms promoted by pirfenidone; red indicates the mechanisms that are inhibited by this drug.

Elucidation of fibrogenic mechanisms is fundamental to identify novel potential therapies, and PFD may be a useful tool in the understanding of these processes, even if further studies are required. In this article, we describe the therapeutic mechanisms of PFD underlying its anti-inflammatory, antifibrotic and antioxidative stress effects, and review the available *in vitro *and *in vivo *models and the clinical trials in which PFD has been used as therapy. We summarize the main molecular mechanisms that are triggered by PFD (Figure 2), and conclude with an examination of fibrosis reduction in the liver (most mechanisms show an equivalent part for fibrosis in lung and renal tissues).

## Three steps in the onset of fibrogenesis

Three important events must be considered to obtain an integral understanding of fibrogenesis: oxidative stress, inflammation and finally fibrosis. The initial biological response to cellular damage includes inflammation and oxidative stress responses. If the insult continues, the anti-inflammatory mechanisms are not sufficient, and a cumulative effect occurs, resulting in greater tissue damage and eventually leading to fibrosis.

Oxidative stress is often associated with fibrogenesis occurring in the liver, lung, arteries and nervous system, because many fibrotic agents stimulate free radical reactions, either directly or through inflammatory stimuli. It is proposed that in fibrotic disorders, a weakened antioxidant defense may define disease susceptibility. In several models, antioxidant supplementation produced a significant reduction in fibrotic progression, reducing the extent of oxidative stress and/or lipid peroxidation [7,8]. It has been reported that in the sequence of events leading to fibrosis, oxidative stress and lipid peroxidation precede, or are concomitant with, HSC activation and collagen deposition [9] From experimental studies, it was shown that NO production was able to prevent both lipid peroxidation and collagen deposition [10,11]. Antioxidants such as silymarin [12,13] α-tocopherol [14,15] silybin [16] and S-adenosylmethionine [17] also produce beneficial effects in fibrotic disorders.

Free radicals appear to modulate the activity of phagocytes and ECM-producing cells. Lipid peroxidation and certain lipid peroxidation products induce genetic overexpression of fibrogenic cytokines, which promote connective tissue deposition, and also induce increased transcription of collagen genes, key molecules in the altered mechanisms of fibrosis. Both events can be downregulated, at least in experimental models, by use of antioxidants [18].

For a better understanding of general antioxidant mechanisms, specialized literature is available [19]. However, even when literature exists on the effects of PFD in activation/repression of antioxidant genes, our knowledge of many processes is incomplete [20,21].

Fibrosis may result from sustained inflammatory processes. Tumor necrosis factor (TNF)-α is a vital component of the inflammatory process, and its aberrant overexpression has been linked to numerous inflammatory states. The anti-inflammatory role of PFD has been exhaustively examined in several models of inflammation. Administration of PFD significantly reduced secreted levels of bioactive and cell-associated TNF-α after stimulation with lipopolysaccharide [22].

Finally, persistent injury results in a chronic wound healing response that eventually leads to fibrosis. This fibrotic response shares common features in multiple organs that can be affected by these disorders. Experimental evidence has indicated PFD as a collagen and TGF-β production blocker, and as an activator of MMPs, thus modulating the fibrogenic pathway [23].

## Organs in which PFD has been tested as an antifibrotic drug

### Lung

A number of studies have been designed to assess the molecular mechanism and the clinical efficacy and safety of PFD when administered to treat lung diseases, including interstitial pulmonary fibrosis, the experimental bleomycin (BL) model of lung fibrosis, and several models of acute lung injury. This group of disorders is characterized by scarring of deep lung tissue, leading to shortness of breath and loss of functional alveoli, thus limiting oxygen exchange. Etiologies include inhalation of inorganic and organic dusts, gases, fumes and vapors, use of medications, exposure to radiation, and development of disorders such as hypersensitivity pneumonitis, coal worker's pneumoconiosis, silicosis and byssinosis (occurs after exposure to cotton dust), among others.

#### *In vitro *models of lung fibrosis and PFD

Recently, it has been proposed that lung fibrosis is caused in part by chronic oxidative stress and inflammation. Increased production of reactive oxygen species (ROS), which leads to lipid peroxidation, oxidation of DNA and proteins, and activation of pro-inflammatory factors, has been observed in several *in vitro *lung models; PFD has been shown to diminish these pathological states. For example, PFD suppressed Hermansky-Pudlak syndrome (HPS)-1, alveolar macrophage cytokine and chemokine secretion *in vitro *in a dose-dependent manner [24] PFD was found to inhibit the responder frequency of T-cell rearrangement (TCR)-stimulated CD4 cell total proliferation *in vitro *and *in vivo*, and the proliferation index of both CD4 and CD8 was reduced. Additionally, PFD inhibited TCR-induced production of multiple pro-inflammatory cytokines and chemokines. Interestingly, there was no change in TGF-β production by purified T cells, and PFD had no effect on the suppressive properties of naturally occurring regulatory T cells [25] In addition, the antifibrotic effect of PFD may be mediated through inhibition of heat shock protein 47, a collagen-specific molecular chaperone, with a resultant reduction in collagen synthesis in lung fibrosis [26].

#### PFD in animal models of lung fibrosis damage

Since the first studies on PFD in lung fibrosis, several recent experimental and clinical trials have provided evidence of its role in decreasing fibrogenesis in the lung. Interstitial pulmonary fibrosis is the result of a wide variety of injuries to the lung, and involves inflammation, increase in cytokine levels, infiltration of immune cells and increase in ECM production in response to proliferating lung fibroblasts [27,28], leading to collagen deposition in the lung interstitium. BL-induced lung damage in rodents resembles this disease [29,30], and is triggered by intratracheal instillation of BL (usually at a dose of 5 to 7.5 U/kg/5 ml) in rodents, producing histological lesions and biochemical changes. Some of the studies with PFD that use this model to assess antifibrotic effects are listed below; in all cases, the dose of PFD was 5% of the weight of the diet fed to the animals throughout the study. The results agreed with those for PFD in other models such as cyclophosphamide-induced fibrosis [31].

Iyer *et al*. [30] demonstrated that PFD retards the progression of an ongoing fibrotic process, and produces a maximum reduction in collagen content of 40% at day 21, through suppression of lung inflammation. In addition, there are decreases in lung lipid peroxidation (a marker of inflammation index), prolyl hydroxylase (PH) activity (an enzyme responsible for post-translational modification of collagen), hydroxyproline content, and pro-collagen I and III mRNA accumulation. However, PFD failed to shown any direct inhibitory effect on PH activity *in vitro*, suggesting that it must be acting at the transcriptional level. As a continuation of this work, Iyer *et al*. [32] measured expression of the TGF-β gene in the lungs after PFD treatment. They observed suppression in the influx of inflammatory cells and macrophages at day 7 post-instillation, and levels of TGF-β mRNA significantly reduced by 33% at 7, 14 and 21 days, as revealed by nuclear run-off studies; TGF-β protein was also suppressed at 14 and 21 days. This effect on TGF-β transcription was also seen in an amiodarone model of lung fibrosis [33].

Furthermore, it was demonstrated that PFD treatment inhibited synthesis of both platelet-derived growth factor (PDGF)-A and -B isoforms by lung macrophages [34], reduced inflammation and suppressed the BL-induced increase in the levels of proteins and TGF-β, and reduced the influx of neutrophils, macrophages and lymphocytes in bronchoalveolar lavage (BAL) fluid at early time points [35]. Using two doses of PFD (30 and 100 mg/kg) three times daily, PFD modulated various pulmonary cytokines at the protein level, and the minimum effective dose was found to be 30 mg/kg three times daily. In the lung, PFD also decreased inflammatory edema, and reduced levels of hydroxyproline, interleukin (IL)-1β, IL-6, IL-12, p40, monocyte chemoattractant protein and interferon (IFN)-γ [36] It also reduced the elevation of lung basic fibroblast growth factor, lung stroma cell derived factor-1α, and IL-18 [36,32] By a different mechanism, induction of arginase, a vital enzyme for collagen synthesis that metabolizes L-arginine to urea and L-ornithine, is altered by PFD treatment, and consequently, levels of collagen contents were reduced in rat lung orthotopic transplants [37]. PFD has also been shown to have a potent anti-TNF-α activity, promoting protection against acute allograft injury [38] and acute lung injury [39] in mice. Additionally, the mechanism of the protective effect of PFD involves a decrease in oxygen radicals in experimental models of acute respiratory distress syndrome [40].

In antigen-induced allergic models, sensitized mice or guinea pigs developed a prominent pulmonary inflammation 24 h after antigen challenge, reflected by a significant increase in the number of recoverable total cells and eosinophils in BAL samples. In both species, pretreatment with PFD (10 and 30 mg/kg) resulted in a dose-dependent inhibition of antigen-induced pulmonary inflammation, which was reflected by a significant decrease in eosinophils and total cells in BAL samples with the 30 mg/kg dose. In a non-allergic model of pulmonary inflammation, rats challenged with intratracheal LPS had a significant increase in neutrophils and total cells in BAL samples, along with significant increases in TNF-α and IL-6. Pre-treatment with PFD (3 and 30 mg/kg) showed a dose-dependent inhibition of the LPS-induced pulmonary inflammation, reflected by a significant decrease in the number of total and neutrophilic cells in BAL samples at both doses. Thus, PFD can inhibit allergic and non-allergic inflammatory cell recruitment, and its pulmonary anti-inflammatory activity is independent of TNF-α inhibition [41].

#### Clinical trials using PFD for lung disease

Idiopathic pulmonary fibrosis (also known as cryptogenic fibrosing alveolitis) is the most common form of interstitial lung disease, and is characterized by chronic progressive pulmonary parenchymal fibrosis. It is a progressive clinical syndrome with unknown etiology; the outcome is invariably fatal as no effective therapy exists.

PFD has been evaluated for its tolerability and usefulness in patients with advanced idiopathic pulmonary fibrosis and other lung diseases. Most reported a few nonsignificant adverse effects (AEs), and found that the drug is generally well tolerated. Raghu *et al*. [42] investigated PFD as oral therapy in consecutive patients with IPF in an open-label study. In this study, 54 patients (mean age 62 years) were monitored for mortality, change in lung function and AEs. The survival rates at 1 and 2 years were 78% and 63%, respectively. Patients whose lung function had deteriorated before enrollment appeared to stabilize after beginning treatment.

In a double-blind, randomized, placebo-controlled trial (RPCT), the effects of PFD were measured by the change in the lowest oxygen saturation by pulse oximetry (SpO_2_) during a 6-minute exercise test. The primary endpoint from baseline to 6 months was not significantly different, but in a subset of patients who maintained a SpO_2 _of > 80% during the 6-minute exercise test at baseline, there was a significant difference in SpO_2 _in the PFD group at 6 and 9 months. PFD also enhanced percentage vital capacity, and episodes of acute exacerbation of IPF occurred exclusively in the placebo group [43].

The disease HPS progressively evolves to fatal pulmonary fibrosis. Treatment with PFD (800 mg three times daily) for up to 44 months in an RPCT changed pulmonary function values. Reduction in predicted forced vital capacity (FVC) each year was 5% slower in 11 PFD-treated patients than in 10 placebo-treated patients. Using data restricted to patients with an initial FVC of > 50% of predicted values, patients in the PFD group lost pulmonary function (FVC, forced expiration volume in 1 second, total lung capacity, and diffusing capacity for carbon monoxide) at a slower rate (> 8%/year) than the placebo group. PFD appears to slow the progression of pulmonary fibrosis in patients with HPS who have significant residual lung function [44].

Given the lack of effectiveness of current therapy in treating patients with IPF, these different points of evidence suggest that PFD might be a useful treatment for this deadly disease.

### Liver

Liver fibrosis occurs as a consequence of ECM accumulation, mainly of collagen types I and III, in response to liver injury. This is triggered by the activation of hepatic stellate cells (HSC), which change to a myofibroblast-like phenotype, with a consequent increase in their synthesis of matrix proteins that characterize fibrosis, such as interstitial collagens [45]. In addition, there is increasing evidence that liver fibrosis is a dynamic pathologic process in which altered matrix degradation may also play a major role. Extracellular degradation of matrix proteins is regulated by MMPs produced by HSC, which in turn are regulated by several mechanisms, including gene regulation (transcription and proenzyme synthesis), cleavage of the proenzyme to an active form, and specific inhibition of activated forms by TIMPs [46].

#### In vitro *models of liver fibrosis and PFD*

In rat HSC, PFD at 1000 μM inhibited PDGF-induced HSC proliferation, without any toxic effects. It also did not affect HSC viability and did not induce apoptosis. The inhibition in cell proliferation was not associated either with variations in PDGF receptor autophosphorylation, or with activation of extracellular signal-related kinase (ERK)1/2 or of the 70 kDa ribosomal S6 kinase (pp70S6K), but PFD was able to inhibit PDGF-induced activation of the Na^+^H^+ ^exchanger involved in PDGF-induced HSC proliferation. PFD also inhibited PDGF-induced protein kinase C activation, type I collagen accumulation and procollagen mRNA expression [47].

In sheep liver microsomes, PFD was found to be ineffective as a superoxide radical scavenger and in decomposing H_2_O_2 _and chelating iron; however, in a deoxyribose degradation assay; PFD was a potent scavenger of hydroxyl radicals, which could be related to its beneficial effects [20].

#### PFD in animal models of liver damage

PFD provides a unequivocal protective anti-inflammatory effect against acute hepatic injury caused by D-galactosamine/LPS in rats by inhibiting elevated TNF levels and IFN-γ, and reducing the induction of inducible nitric oxide synthase (iNOS)/nitric oxide (NO) [48], partly through the inhibition of nuclear factor κB activation [49]. PFD also inhibits production of cytokine-induced neutrophil chemoattractant and macrophage inflammatory protein-2, (induced by IL-1β at posttranscriptional steps in hepatocytes) in the process of neutrophil recruitment and activation [50]. Previous data from our group [51] showed that PFD is an effective antifibrotic drug in two different experimental models of fibrosis (basal laminar deposits (BLD) and chronic intoxication by carbon tetrachloride (CCl_4_)), significantly decreasing levels of alanine aminotransferase (ALT), aspartate aminotransferase and alkaline phosphatase compared with saline-treated animals. Fibrotic areas reduced by 50% in 4-week BDL rats, and by 70% in a CCl_4 _model, along with hydroxyproline levels. The number of activated HSC decreased, and there was a reduction in gene expression of collagens I, III and IV, TGF-β1, Smad-7, TIMP-1 and plasminogen activator inhibitor (PAI)-1.

It has been shown that PFD maintains its antifibrotic properties when administered after hepatic damage has already occurred. Rats treated with dimethylnitrosamine 10 mg/kg for 5 weeks received a liquid diet containing 0.5% PFD starting from the third week. The PFD treatment reduced the degree of liver injury, as determined by ALT values and necroinflammatory score, which was associated with reduced HSC proliferation and collagen deposition. Treatment with dimethylnitrosamine produced a fold increase in transcript levels of TGF-β1, TIMP-1 and MMP-2 of seven, seven, four and 15, respectively. PFD downregulated the elevated levels of these transcripts by 50 to 60%, which was associated with a 70% reduction in collagen deposition and downregulation of TGF-β1 and of MMP-2 mRNA, the two substances mainly implicated in the degradation of normal ECM [52].

In bile duct ligation and CCl_4 _models of liver cirrhosis, PFD treatment caused a reduction in inflammation and in hepatic enzymes and bilirubin concentrations. It also downregulated TGF-β1 and collagen I-α(COL1A1) genes [21]. In addition, in this paper, our group demonstrated the potent role of PFD as an antioxidant *in vivo *compared with a well-known broad-spectrum antioxidant such as diphenyleneiodonium. The antioxidant capacity of PFD produced a 28% and 30% reduction, respectively, in nitrite and malonyldealdehide concentrations in the bile duct ligation model, and 52% and 38% in the CCl_4 _model. Furthermore, PFD downregulated gene expression of superoxide dismutase (SOD), catalase (CAT) and iNOS. The functional activity of SOD and CAT also decreased after PFD administration, raising the possibility of using PFD for diseases accompanied by oxidative stress.

#### Clinical trials using PFD for liver diseases

Owing to the wide range of etiologies that lead to fibrogenesis in the liver, specific and effective therapies for each kind of fibrosis remain elusive [53]. Furthermore, the effectiveness of PFD in many cases depends on inherited genetic polymorphisms that increase the risk of developing advanced fibrosis in patients with established liver fibrosis [54]. Despite this, in a pilot clinical trial evaluating the safety and efficacy of PFD, 15 patients with established advanced liver disease caused by chronic hepatitis C virus infection had improvements in liver histology (necrosis, inflammation, steatosis, fibrosis and cell regeneration) 12 months after oral PFD therapy (1200 mg/daily). Liver cell regeneration was detected in 70% of patients with differing degrees of anti-proliferating cell nuclear antigen as measured by immunostaining. Fibrosis was reduced in 30% of patients by the end of the 12-month treatment, and mRNAs coding for profibrogenic molecules such as COL1A1, TGF-β1 and TIMP-1 were markedly downregulated by the end of treatment. Quality of life, as measured by the Short Form (SF)-36 questionnaire imseen markedly in all patients [55].

### Kidney

#### PFD and *in vitro *models of renal fibrosis

Fibroblasts are activated in tubulointerstitial injury and their presence is a marker of disease progression. A well-characterized model of experimental renal disease is the unilateral ureteral obstruction (UUO) which culminates in tubulointerstitial fibrosis.

Cortical fibroblasts isolated from kidneys 3 days after UUO were exposed to increasing PFD concentrations, which produced a decrease in cell proliferation, α-smooth muscle actin and connective tissue growth factor protein expression, although synthesis of collagen was unaffected by PFD [56].

#### PFD in animal models of renal fibrotic damage

Using five-sixths nephrectomy rat model, the effect of PFD on the progression of chronic renal failure was examined. PFD-treated rats had inhibition of TGF-β1, type IV and I mRNA collagen expression [57].

With the UUO model, rats had upregulation of mRNA for collagen types I and IV, MMP-2 and TGF-β1. In addition, a progressive increase in hydroxyproline content was observed in the post-obstructed kidney despite the release of obstruction, but these increases were suppressed by PFD. Thus PFD can attenuate both renal fibrosis and renal damage in this model, and therefore could be clinically useful for preventing progressive, irreversible renal failure [58].

One more animal model in which the antifibrotic properties of PFD were observed was the model of chronic nephrotoxicity induced by ciclosporin (CsA), which is characterized by tubulointerstitial fibrosis. Treatment with PFD ameliorated CsA-induced fibrosis by about 50%. PFD was associated with a decrease in TGF-β1 expression, which in turn was associated with a decrease in matrix deposition [59].

Additional proof of reduction in ECM by PFD was observed in mouse mesangial cells, in which PFD decreased TGF-β promoter activity, reduced TGF-β protein secretion, and inhibited TGF-β-induced Smad2-phosphorylation, 3TP-lux promoter activity and generation of ROS. In addition, PFD treatment significantly reduced mesangial matrix expansion and expression of renal matrix genes. Thus, the renoprotective and antifibrotic PFD effects could be related, at least in part, to its inhibition of RNA processing [60].

Streptozotocin (STZ)-induced diabetic rats treated with PFD and spironolactone showed both reversal in deposition of major ECM proteins, collagen and fibronectin, and a number of functional changes. Fibrosis leads to chronic impairment of cardiac and renal function, thus reversal of existing fibrosis may improve function and survival. Short-term treatment with PFD and spironolactone reversed cardiac and renal fibrosis and attenuated the increased diastolic stiffness, but without normalizing cardiac contractility or renal function in STZ-induced diabetic rats [61].

The ability of PFD to reverse markers of renal dysfunction in rats was also tested. Tacrolimus-induced nephrotoxicity is thought to contribute to renal allograft dysfunction and subsequent failure, a process that is underpinned by alterations in mRNA expression of genes involved in matrix metabolism. PFD caused a decrease in collagen III and TIMP-1 mRNA expression, suggesting that it could attenuate the limited fibrotic potential of tacrolimus [62].

PFD and the angiotensin II type I receptor antagonist candesartan cilexetil, given alone or in combination, were tested in rats with chronic antiglomerular basement membrane glomerulonephritis (anti-GBM GN). The combination of both agents produced an improvement in adsorption droplets and proteinuria in the glomeruli, and cortical collagen I mRNA expression was also significantly decreased. Rats treated with PFD had blood pressure values similar to control rats. Thus, the beneficial effects of PFD on morphological changes in anti-GBM GN were comparable with those of candesartan, and these results suggest an additive effect of combination treatment [63].

In a model of spontaneous progressive glomerulosclerosis using FGS/Kist mice, PFD was evaluated for the prevention of renal fibrosis; proteinuria levels were lower in the PFD group compared with the control diet group The sclerosis scores of the PFD groups at 3 months were also reduced. There was no significant difference between the PFD and control diet groups after treatment for 1 or 2 months, but there was a significant difference after treatment for 3 months, suggesting that long-term administration of PFD is required to suppress the progression of glomerulosclerosis and improve renal function in the FGS/Kist mice [64].

In a vanadate-induced kidney fibrosis model in rats, the antifibrotic effects of PFD were also seen. Treatment with PFD reduced vanadate-induced increases in kidney weight, RNA content and hydroxyproline levels. Histological evaluation revealed that the severity of the lesions in the vanadate-treated group was 'moderate to severe' before treatment with PFD; after treatment with PFD for 41 days, the severity decreased to 'mild'. The collagen content of the kidney was also reduced after PFD treatment [65].

Finally, PFD produced a modulation of apoptosis mediators in a chronic CsA-induced nephrotoxicity animal model. PFD reduced the number of apoptosis-positive cells induced by CsA. In addition, PFD downregulated mRNA expression of CsA-induced p53 and Fas-ligand and increased that of Bcl-xL, which had previously been reduced by CsA. PFD significantly downregulated caspase 3 expression, mostly on renal tubular epithelial cells. Because apoptosis could partly explain the loss of cells associated with fibrosis, the influence of PFD on apoptosis-regulatory genes to cause a reduction in apoptosis may explain some of its antifibrotic properties [66].

#### Clinical trials using PFD for renal diseases

PFD was tested in patients with focal segmental glomerulosclerosis. The monthly change in estimated glomerular filtration rate (GFR) was compared between baseline and after treatment. Patients received angiotensin antagonist therapy if tolerated. In total, 18 patients completed a median of 13 months of PFD treatment; the monthly change in GFR improved from a median of -0.61 ml/min per 1.73 m^2 ^at baseline to -0.45 ml/min per 1.73 m^2^., a median improvement in the rate of decline of 25%. It was concluded that PFD slows renal function decline in patients with focal segmental glomerulosclerosis [67].

Details of the clinical trials that used pirfenidone for various fibrotic diseases are listed in Table 1.

**Table 1:** Summary of the clinical trials that used pirfenidone for fibrosis-related diseases

Disease	Dose and time	Type of study	Number of patient	Effects	Reference
Interstitial pulmonary fibrosis	3600 mg/daily for 2 years	Open-label study	54	Increase in 1 and 2 year survival. Stabilized lung function.	[42]
Interstitial pulmonary fibrosis	600 mg three times daily for 12 months	Double-blind, randomized, placebo- controlled trial	107	Improvement in SpO_2 _during a 6-minute exercise test. No episodes of acute exacerbation of IPF in PFD group.	[43]
Hermansky-Pudlak syndrome	800 mg three times daily for 44 months	Randomized, placebo-controlled trial	11	Loss of pulmonary functions occurred at a slower rate.	[44]
HCV related liver disease	1200 mg/daily for 12 months	Open-label pilot study	15	30% of patients had less fibrosis after treatment. Downregulation of Col I, TGFβ and TIMP-1. Improvement in quality of life (SF-36 test).	[55]
Focal segmental glomerulosclerosis	800 mg three times daily for 5 to 37 months	Open-label pilot study	18	Slowed renal function decline; improvement of 25%	[67]
Hypertropic scars	PFD 8% gel for 6 months	Open-label pilot study	33	Improvement in the Vancouver scar score in 66.6% of patients.	[70]

HCV = hepatitis C virus; PFD = pirfenidone; SF = Short Form; SpO_2 _= lowest oxygen saturation by pulse oximetry; TGF = transforming growth factor; TIMP = tissue inhibitor of metalloproteinase.

### Fibrogenesis in other tissues and organs

It was also observed that PFD reduces AEs such as capsule contracture in mammary implants in an animal model, Gancedo *et al*. [68] observed a reduction in capsule thickness around submmamary tissue, along with a decrease in fibroblast-like cell proliferation, and recruitment and infiltration of inflammatory cells.

It has been shown that PFD reduces keloid formation in an animal model. A keloid is a type of scar with mainly type I and some type III collagen, which results in an overgrowth of tissue at the site of a healed skin injury. Keloids should not be confused with hypertrophic scars, which are raised scars that do not grow beyond the boundaries of the original wound. In athymic nude mice (nu-nu), PFD significantly reduced the weight of keloid implants weight compared with control implants at 60 and 90 days after implantation. PFD may cause increased degradation and absorption of keloid tissue [69].

Our own clinical data has demonstrated that a scar reduction gel (Kitoscell™; Cell Therapy and Technology, Mexico City, Mexico) with 8% PFD topically administered during a 6-month period led to resolution of hypertrophic scars acquired after burns in pediatric patients. There was a significant improvement after treatment in all patients: 27.27% had a decrease of 55%; 66.66% a decrease of 30 to 45%, and the remainder a decrease of ≤ 30%, according to the Vancouver scar scale [70].

Pesce E [71] compared the antirheumatic effect of PFD with a positive control drug, oxyphenbutazone, which is used in patients with rheumatoid arthritis (RA), in a double-blind clinical trial in humans. It was found that PFD was more effective than oxyphenbutazone in providing relief from arthritic pain. In addition, a greater number of patients reported a favorable response to oral PFD than to oral oxyphenbutazone. There were no significant differences between the PFD and oxyphenbutazone groups in the number of patients who dropped out from the trial or who tolerated the drugs for the 21 days of the trial, indicating that PFD is a potential new therapy for the management of RA, with few or no AEs, unlike the steroidal and non-steroidal anti-inflammatory drugs that are frequently used for this chronic debilitating disease.

Currently, there are no approved treatments for *s*econdary progressive MS that stabilize or reverse the neurological disabilities associated with this disease. Oral PFD was found to stabilize and overcome the symptoms of secondary progressive MS in a phase II double-blind RPCT in patients who had advanced secondary progressive MS that had been present for at least 12 months. After 1 month of treatment with PFD, patients had improvement in their Scripps Neurological Rating Scale (SNRS) scores, and scores remained significantly improved for 3, 6 and 12 months compared with baseline SNRS scores. By contrast, the SNRS scores of patients on oral placebo were not significantly improved compared with baseline scores [72].

## Conclusions

The advantages of PFD clearly exceed any possible AE ascribable to this drug. It obviously had powerful antifibrotic properties, as it can reduce oxidative, inflammatory and pro-fibrogenic markers. As many therapeutic agents target only one of these types of markers, but still produce a reduction in, PFD seems to be a particularly valuable drug. Thus, this agent could be as a promising drug not just in animal models, but also in clinical studies of fibrotic diseases, and eventually as a therapy for such diseases.

## Competing interests

The authors declare that they have no competing interests.

## Authors' contributions

JM-B carried out the *in vivo *and *in vitro *liver research with PFD, and contributed to the manuscript writing. AS-R wrote the lung section and made style and syntax changes to the manuscript. JN-P revised the manuscript, JA-B led the group working with PFD and oversaw the manuscript writing and style. All authors read and approved the final manuscript.
